# TGFβ1 enhances *MAD1 *expression and stimulates promoter-bound Pol II phosphorylation: basic functions of C/EBP, SP and SMAD3 transcription factors

**DOI:** 10.1186/1471-2199-12-9

**Published:** 2011-02-23

**Authors:** Nadine Hein, Kan Jiang, Christian Cornelissen, Bernhard Lüscher

**Affiliations:** 1 Institute of Biochemistry and Molecular Biology, Medical School, RWTH Aachen University, 52057 Aachen, Germany; 2 Peter MacCallum Cancer Center, Growth Control and Differentiation Program, Laboratory of Growth Control, Melbourne, Australia; 3 Laboratory of Cellular Oncology, Center for Cancer Research, National Cancer Institute, National Institutes of Health, Bethesda, MD, USA

## Abstract

**Background:**

The MAD1 protein, a member of the MYC/MAX/MAD network of transcriptional regulators, controls cell proliferation, differentiation and apoptosis. MAD1 functions as a transcriptional repressor, one direct target gene being the tumor suppressor *PTEN*. Repression of this gene is critical to mediate the anti-apoptotic function of MAD1. Under certain conditions it also antagonizes the functions of the oncoprotein MYC. Previous studies have demonstrated that *MAD1 *expression is controlled by different cytokines and growth factors. Moreover we have recently demonstrated that the *MAD1 *promoter is controlled by the cytokine granulocyte colony-stimulating factor (G-CSF) through the activation of STAT3, MAP kinases and C/EBP transcription factors.

**Results:**

We observed that in addition to G-CSF, the cytokine transforming growth factor β (TGFβ1) rapidly induced the expression of *MAD1 *mRNA and protein in promyelocytic tumor cells. Moreover we found that C/EBP and SP transcription factors cooperated in regulating the expression of *MAD1*. This cooperativity was dependent on the respective binding sites in the proximal promoter, with the CCAAT boxes being bound by C/EBPα/β heterodimers. Both C/EBP and SP transcription factors bound constitutively to DNA without obvious changes in response to TGFβ1. In addition SMAD3 stimulated the *MAD1 *reporter, cooperated with C/EBPα and was bound to the core promoter region. Thus SMAD3 appears to be a potential link between TGFβ1 signaling and C/EBP regulated promoter activity. Moreover TGFβ1 stimulated the phosphorylation of polymerase II at serine 2 and its progression into the gene body, consistent with enhanced processivity.

**Conclusions:**

Our findings suggest that C/EBP and SP factors provide a platform of transcription factors near the core promoter of the *MAD1 *gene that participate in mediating signal transduction events emanating from different cytokine receptors. SMAD3, a target of TGFβ1 signaling, appears to be functionally relevant. We suggest that a key event induced by TGFβ1 at the *MAD1 *promoter is the recruitment or activation of cofactors, possibly in complex with C/EBP, SP, and SMAD3 transcriptional regulators, that control polymerase activity.

## Background

The MYC/MAX/MAD network of transcriptional regulators is essential to control many aspects of cell physiology [1]. MYC was originally identified as oncogene in several different chicken retroviruses. Subsequently the three human MYC genes, MYC, MYCN and MYCL were found deregulated in the large majority of human tumors [2]. The potent capacity of MYC to transform cells has also been supported by a large number of studies in both primary cells and established cell lines and in animal models. Central to the ability to transform cells is MYC's function as transcriptional regulator in controlling the expression of a large number of target genes. This explains, at least in part, the broad biological activities associated of MYC [3,4]. The functions of MYC in gene expression control depend largely on its interaction with MAX, the central component of the MYC/MAX/MAD network.

MAD proteins are alternative binding partners of MAX [5]. Six different MAD proteins have been identified. MAD1-4 are highly related, while MNT and MGA are considerably larger multi-domain proteins. Similar to MYC, the MAD proteins are transcriptional regulators, with MAD1-4 primarily described as repressors. Unlike MYC proteins, the MADs have not been linked to human diseases, in particular they appear not to be tumor suppressors as one might have expected. For MAD1-4 the reason for their apparent lack to function as tumor suppressors may be in part due to their broad and overlapping expression pattern, suggesting that more than one MAD family member would need to be inactivated in tumors [5]. In addition, MAD proteins, best studied for MAD1, have anti-apoptotic activity and thus may antagonize the pro-apoptotic functions of MYC proteins [6-8]. This activity of MAD proteins may be indispensable for tumor development. In support, one of the few MAD1 target genes that has been identified is the tumor suppressor gene *PTEN *[8]. MAD1, which functions primarily as a transcriptional repressor by recruiting histone deacetylase-containing complexes [9-12], represses the *PTEN *promoter directly [8]. This contributes to the anti-apoptotic phenotype elicited by MAD1. The analysis of granulocytes from mice lacking *Mad1 *revealed increased sensitivity to pro-apoptotic conditions [6], further supporting the view that MAD1 protects cells from different apoptotic stimuli.

In addition to the anti-apoptotic function, MAD1 has been suggested to control proliferation and differentiation antagonistically to MYC [5]. Indeed the unscheduled expression of MAD1 interferes with cell proliferation and the lack of *Mad1 *results in a differentiation defect of granulocytes [6,7,13-15]. During the studies to elucidate the functions of MAD1 in proliferation and differentiation, it had been noted early on that the expression of the *MAD1 *gene is highly regulated, generally reciprocal to the regulation of *MYC *genes [5]. Moreover MAD1 expression is directly downregulated by MYC (NH and BL, unpublished observations). In particular several differentiation inducing agents, including transforming growth factor β (TGFβ), retinoic acid, and granulocyte-colony stimulating factor (G-CSF), were identified as stimulators of *MAD1 *expression [16-20]. These findings led us to address the question how the *MAD1 *promoter is organized and how signals of these differentiation factors control gene expression. The *MAD1 *promoter contains a CpG island as part of a roughly 400 bp proximal promoter region highly conserved between humans and rodents [17]. This region is responsive to G-CSF, integrating signals transduced from the G-CSF receptor by STAT3 and by the RAS-RAF-ERK pathway. This regulation of the *MAD1 *promoter by G-CSF is in agreement with the described role of this cytokine and of Mad1 in the control of granulocyte differentiation and survival [6].

Cytokines of the TGFβ family have broad activities in controlling cell physiology, including proliferation, differentiation and survival [21-23]. TGFβ signals through TGFβ type II and I receptors with Ser/Thr kinase activity, thereby activating SMAD proteins, in particular SMAD2 and 3 in combination with SMAD4. These proteins translocate to the cell nucleus and form complexes with additional molecules to control the expression of target genes [24]. We have shown previously that the phorbol ester TPA and TGFβ activate the expression of *MAD1 *in U937 and in HaCaT keratinocytes, respectively [18,19]. In both systems a substantial increase in mRNA expression was observed by 90 min, suggesting that the induction was direct. Different kinetics of *MAD1 *induction were observed in a clone of U937 promyelocytes that stably express a viral version of MYC (U937-myc6). In these cells a weak induction was observed in response to TGFβ by 8 hrs, possibly as a result of constitutive MYC expression [20]. To understand in more detail how TGFβ1 regulates *MAD1 *gene expression, we addressed how this cytokine affects *MAD1 *promoter activity. It appears that TGFβ1 stimulates *MAD1 *through elements proximal to the core promoter.

## Results and Discussion

### Rapid activation of *MAD1 *by TGFβ

**Figure 1:**
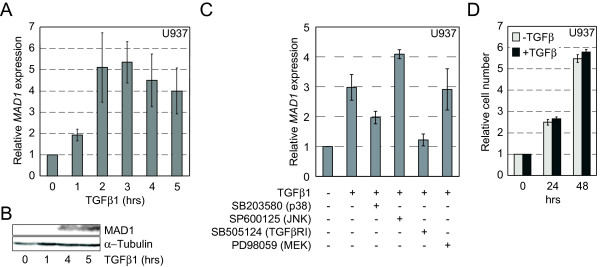
**Induction of *MAD1 *mRNA and protein expression in response to TGFβ1 in U937 cells**. **A**. Exponentially proliferating U937 promyelocytes were treated with 2.5 ng/ml of TGFβ1. mRNA was purified at the indicated time points. The kinetics of *MAD1 *expression in response to TGFβ1 were analyzed by quantitative RT-PCR. The mean values ± SD of three independent experiments are shown. **B**. Whole cell extracts of U937 cells treated as in panel A were generated at the indicated time points and analyzed for endogenous MAD1 protein by western blotting. Tubulin served as loading control. **C**. U937 cells were pre-treated with the TGFβ receptor I inhibitor SB505124 (5 μM) for 40 min prior to addition of TGFβ1. *MAD1 *expression was assayed by quantitative RT-PCR 2 hrs after TGFβ1 stimulation. The displayed data are mean values ± SD of three independently performed experiments. **D**. U937 cells were initially diluted to a density of 10^5 ^cells/ml and incubated with 2.5 ng/ml TGFβ1 over a period of 48 hrs. The number of cells was determined at the indicated time-points by CASY measurements.

During cell proliferation and differentiation, the *MAD1 *gene is regulated by multiple signaling pathways. One of the regulatory cytokines is TGFβ1, which is known to induce MAD1 in keratinocytes and in U937-myc6 promyelocytes [19,20]. To further evaluate the role of TGFβ1 in regulating *MAD1*, we performed time course experiments. TGFβ1 rapidly activated *MAD1 *mRNA expression in U937 cells (Figure 1A). In parallel, MAD1 protein became detectable within 4 hrs of TGFβ1 stimulation (Figure 1B). Thus the induction of MAD1 protein follows closely the up-regulation seen at the mRNA level. The induction of *MAD1 *expression was dependent on the TGFβ receptor (TGFβR) since the TGFβRI inhibitor SB505124 blocked *MAD1 *activation (Figure 1C). Moreover inhibition of the MAPK p38 resulted in a partial inhibition of *MAD1 *expression in response to TGFβ1, whereas the inhibition of JNK or ERK kinases did not repress *MAD1 *expression (Figure 1C). The activities of the inhibitors were verified by analyzing the phosphorylation of the relevant kinases (data not shown). These findings indicate that TGFβ1 may signal by different pathways to the *MAD1 *promoter. Indeed the TGFβR is known to activate several different signaling cascades in addition to SMAD transcription factors, including different MAP kinases and the PI3K-AKT pathway [25].

MAD1 has been demonstrated to interfere with cell proliferation in some cell types [7,14]. Therefore we measured whether the induction of MAD1 by TGFβ1 affected the proliferation of U937 tumor cells. However the early TGFβ1-stimulated induction of MAD1 was not sufficient to block U937 proliferation (Figure 1D), similar to the observations made in U937-myc6 cells [20]. Our findings suggest that tumor cells like U937 have the possibility to bypass at least transiently the repressive function of MAD1 in cell proliferation.

### C/EBPα/β heterodimers bind constitutively to the *MAD1 *promoter

**Figure 2:**
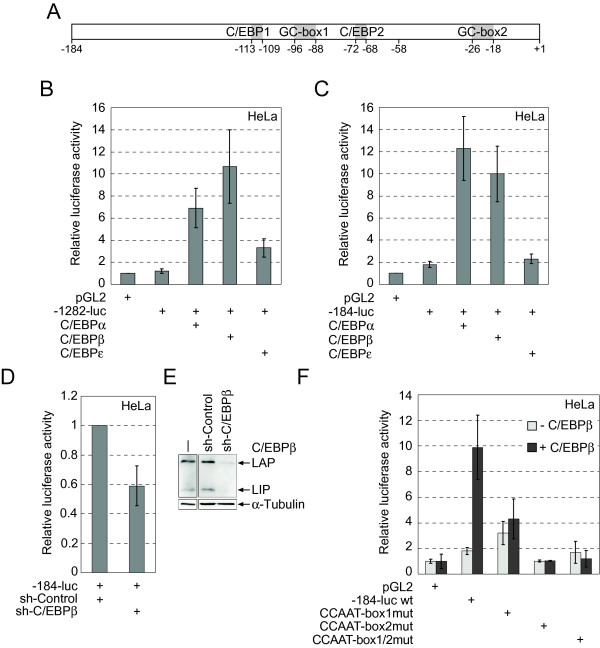
***MAD1 *promoter reporter gene constructs are regulated by C/EBP transcription factors**. **A**. Schematic representation of the position of the CCAAT box (half-sites) and GC box motifs within the *MAD1 *promoter region. The major transcriptional start site is indicated with +1. **B**. and **C**. HeLa cells were co-transfected with the -1284 to +248 or the -184 to +248 reporter gene constructs, respectively, and plasmids encoding the three indicated C/EBP proteins. The promoter-less pGL2 construct served as negative control and its activity was set as 1. The luciferase measurements were normalized to β-galactosidase activities expressed from a co-transfected plasmid. **D**. Reporter gene assays were performed in HeLa cells by co-transfecting the -184 to +248 reporter gene construct and a pSuper-based shRNA targeting C/EBPβ or a control shRNA. Luciferase activity was determined 2 days after transfection. **E**. The efficiency of the shRNA targeting C/EBPβ was examined in HeLa cells by immunoblotting. C/EBPβ-specific antibodies were used to detect the two major isoforms of endogenous C/EBPβ proteins, LAP and LIP, in whole cell extracts. **F**. The C/EBPβ-mediated relative activation of the indicated *MAD1 *promoter constructs were analyzed in reporter gene assays performed in HeLa cells as described above.

The *MAD1 *promoter does not contain any obvious SMAD binding sites in the proximal region. Indeed a recent study suggested that SMAD2/3 stimulate *MAD1 *expression independent of SMAD4, possibly through an indirect mechansism [26]. Moreover it has been found that SMAD proteins may interact with C/EBP transcription factors to control gene expression [27]. Since we have shown previously that C/EBPs control the transcription of *MAD1 *in response to the cytokine G-CSF in RK13 rabbit epithelial cells [17], we addressed the role of C/EBP transcription factors in human cells. Transient transfection experiments in HeLa cells demonstrated that C/EBPα and β, and to a lesser extend C/EBPε, were able to stimulate -1282 to +248 and -184 to +248 (relative to the major transcriptional start site) *MAD1 *promoter reporter gene constructs (Figure 2A-C). Moreover knockdown of C/EBPβ reduced *MAD1 *promoter reporter gene activity, suggesting that its expression is controlled by endogenous C/EBPβ (Figure 2D and 2E). This appears to be a direct effect since the mutation of the two CCAAT box-like sequences in the promoter proximal region affected the sensitivity to C/EBPβ (Figure 2A). Deletion of box1 reduced, while deletion of either box2 or both elements together eliminated promoter activity in response to C/EBPβ (Figure 2F). Together these findings demonstrate that, similar to the observations in RK13 cells, C/EBPs also control *MAD1 *expression in human cells.

**Figure 3:**
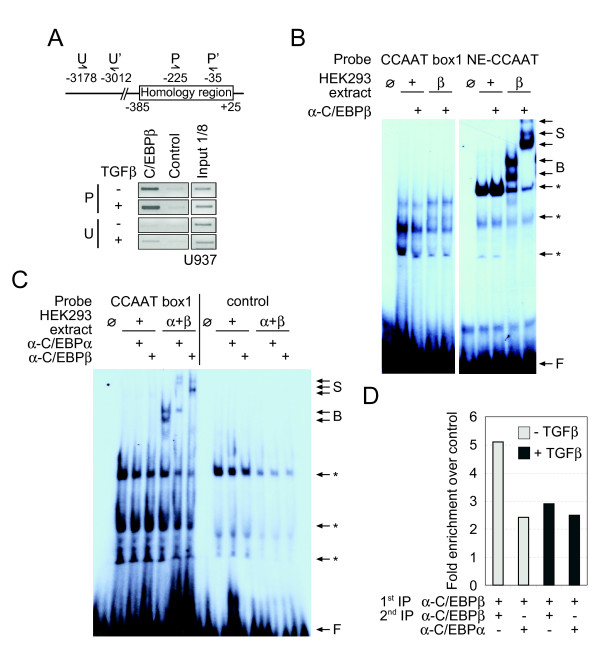
**C/EBPβ binds to the chromatin embedded *MAD1 *promoter in cells**. **A**. Schematic presentation of the *MAD1 *promoter relative to the major transcriptional start site with the homology region as defined in [17]. The positions of primer sets used for ChIP PCR are given (upper panel). U937 cells were used in ChIP assays to investigate the binding of C/EBPβ to the *MAD1 *promoter in response to TGFβ1. The cells were treated with 2.5 ng/ml TGFβ1 for 1.5 hrs prior to crosslinking and immunoprecipitation, and the purified DNA was analyzed by PCR with the indicated primers. The displayed experiment is representative of three independent experiments with similar outcome. **B**. C/EBPβ was expressed in HEK293 cells. Whole cell extracts were generated and incubated with [^32^P]-radiolabelled CCAAT box1 oligonucleotids derived from the *MAD1 *or the neutrophil elastase promoter (NE-CCAAT). Supershift experiments with C/EBPβ specific antibodies are indicated. The complexes were analyzed by EMSA and detected by autoradiography. Ø, indicates lanes without cell extracts; +, whole cell extracts of control transfected HEK293 cells; β, whole cell extracts of HEK293 cells transiently expressing C/EBPβ. **C**. The C/EBPα/β heterodimer DNA-binding activity to CCAAT box1 was determined by EMSA. HEK293 cell extracts overexpressing C/EBPα and C/EBPβ were generated as in panel B and incubated with radiolabelled CCAAT box1. Supershift experiments were performed with C/EBPα- or C/EBPβ-specific antibody. For control an irrelevant oligonucleotide was used. **D**. Re-ChIP experiment using C/EBPα- and C/EBPβ-specific antibodies in U937 cells treated with or without TGFβ1 for 1.5 hrs. The first immunoprecipitation was performed with antibodies specific for C/EBPβ. The protein-DNA complexes were then released and subjected to a second immunoprecipitation using C/EBPα or C/EBPβ specific antibodies. The isolated DNA was analyzed by quantitative PCR with the primer set P (panel A) amplifying a portion of the homology region of the *MAD1 *promoter. The signal intensities obtained with the specific immunoprecipitations were normalized to samples generated with control antibodies. For all panels one representative of typically at least three independent experiments are shown.

To address whether TGFβ1 might affect C/EBP binding to the *MAD1 *promoter, ChIP experiments were performed. Specific C/EBPβ binding to the core-promoter region was observed, whereas only weak interaction with a more distal promoter region could be detected (Figure 3A). C/EBPβ was found at the *MAD1 *promoter prior to TGFβ1 signaling (Figure 3A). Stimulation by TGFβ1 did not result in altered binding. Thus C/EBP proteins interact with the promoter independent of TGFβ1 signaling. The binding of C/EBP proteins to the CCAAT box motifs, both appear only to be half-sites [17], was further evaluated using electrophoretic mobility shift assays (EMSA). Neither of the two half-sites (i. e. CCAAT box1 and CCAAT box2) was bound by C/EBPα or C/EBPβ homodimers alone when expressed in HEK293 cells (Figure 3B and data not shown). For control efficient and specific binding of C/EBPβ and C/EBPα to a CCAAT box of the neutrophil elastase gene was measurable, as reported previously [28] (Figure 3B and data not shown). Since the findings using ChIP and EMSA were contradictory, we expanded the EMSA experiments by evaluating the binding of C/EBPα/β heterodimers. In contrast to the homodimers, the heterodimeric C/EBP complexes interacted with the CCAAT box1 (Figure 3C) and less well with CCAAT box2 (data not shown). The presence of a heterodimeric complex at CCAAT box1 was verified using C/EBPα and β specific antibodies. Both antibodies were able to supershift the complexes observed, further validating that C/EBPα/β heterodimers were able to bind to the *MAD1 *promoter. To address whether the chromatin embedded *MAD1 *promoter was bound by C/EBPα/β heterodimers, re-ChIP experiments were performed by immunoprecipitating first chromatin-bound C/EBPβ. The bound material was released and re-immunoprecipitated with antibodies specific for either C/EBPα or C/EBPβ (second immunoprecipitation) in comparison to a control (Figure 3D). The specific signals obtained with both C/EBP antibodies suggested that indeed the *MAD1 *promoter was occupied by C/EBPα/β heterodimers. Again this was largely independent of TGFβ signaling (Figure 3D).

### SP transcription factors bind to the MAD1 promoter independent of TGFβ signaling

**Figure 4:**
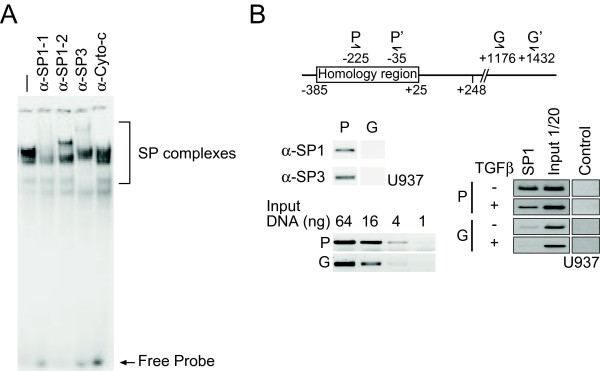
**Constitutive SP1 and SP3 binding to the chromatin embedded *MAD1 *promoter**. **A**. Nuclear extracts of U937 cells were incubated with radiolabelled GC box1 and supershift experiments were performed with SP1 (α-SP1-1, SC-59; α-SP1-2, obtained from G. Suske) and SP3 specific antibodies. The EMSA was performed as described in the legend to Figure 3. **B**. Schematic representation of the positions of primer sets used for ChIP PCR relative to the major transcriptional start site within the *MAD1 *gene (upper panel). U937 cells were treated with 2.5 ng/ml TGFβ1 for 1.5 h, cross-linked and examined for SP1 and SP3 binding by ChIP. The isolated DNA was analyzed by PCR using the indicated primer sets.

In addition to CCAAT boxes, the proximal promoter region of the *MAD1 *gene contains 2 prominent GC boxes (Figure 2A). To test whether SP proteins can bind to either of these two GC boxes, we performed EMSA and ChIP experiments. Prominent binding to an oligonucleotide spanning GC box1, which is flanked by the two CCAAT boxes, was observed in EMSA experiments using U937 cell extracts (Figure 4A). Binding to GC box2 was weaker (data not shown). Supershift experiments using specific antisera indicated that both SP1 and SP3 proteins bind to GC box1 (Figure 4A). Moreover both proteins bound constitutively to the chromatin embedded proximal *MAD1 *promoter that contains GC box1 and no change in response to TGFβ1 was measurable (Figure 4B and data not shown). Similarly the binding of SP1 and SP3 to the *MAD1 *promoter was not affected by G-CSF (data not shown), indicating that these transcription factors as well as C/EBP proteins are constitutively interacting with the *MAD1 *promoter.

### C/EBP and SP transcription factors cooperate in stimulating the *MAD1 *promoter

**Figure 5:**
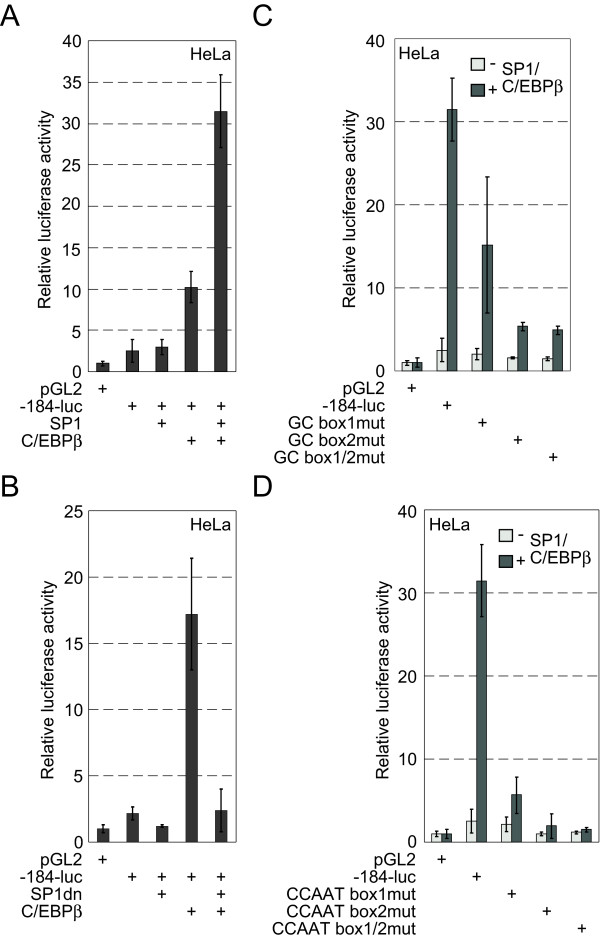
**SP1 and C/EBPβ cooperate in *MAD1 *promoter reporter gene activation**. **A**. HeLa cells were co-transfected with the -184 to +248 reporter gene construct and C/EBPβ and/or SP1 expression vectors as indicated. **B**. A dominant negative SP1 mutant (SP1dn) was co-expressed with C/EBPβ in HeLa cells and the activity of the -184 to +248 reporter gene construct was determined. **C**. and **D**. HeLa cells were transiently transfected with the indicated expression plasmids and the -184 to +248 reporter gene construct or reporter constructs with the indicated mutations in the identified response elements.

Since the CCAAT and GC boxes are in close proximity within the *MAD1 *promoter (Figure 2A), we addressed whether SP1 and C/EBPβ were able to cooperate on *MAD1 *reporter gene constructs. While SP1 alone had no effect on the expression of the reporter gene, it substantially stimulated C/EBPβ-dependent expression (Figure 5A). This observation was further validated by expressing a dominant negative form of SP1 (SP1dn), which lacks the transactivation domain. SP1dn repressed efficiently C/EBPβ-induced *MAD1 *promoter reporter gene expression (Figure 5B). The cooperative effect of SP1 and C/EBPβ was dependent on the GC and CCAAT boxes (Figure 5C and 5D). Together these findings suggest that SP and C/EBP proteins bind to the proximal *MAD1 *promoter and cooperate in activating the *MAD1 *promoter.

### SMAD3 interacts with and activates the *MAD1 *promoter dependent on C/EBP and SP binding sites

**Figure 6:**
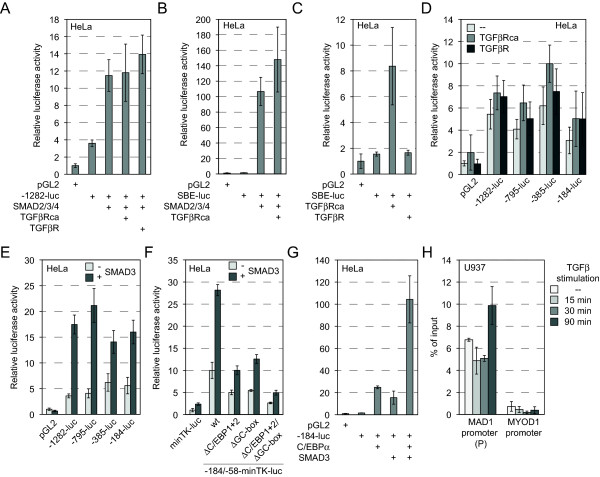
**Influence of SMAD proteins and TGFβ1 receptor signaling on MAD1 promoter activity**. **A**. HeLa cells were co-transfected with the -1282 to +248 reporter gene construct and SMADs and/or TGFβRca or TGFβR expression vectors as indicated. For control the pGL2 reporter was measured. **B and C**. As in A except that the SMAD binding element (SBE) reporter gene was used. **D**. The effect of TGFβRca and TGFβR was measured on different reporter gene constructs containing different fragments of the *MAD1 *promoter in HeLa cells (as indicated). **E**. As in panel D except that the effect of SMAD3 was determined on different fragments of the *MAD1 *promoter. **F**. The effect of SMAD3 was determined on the -184 to -58 fragment of the *MAD1 *promoter. This fragment contains the relevant C/EBP half sites and the GC box that interacts with SP factors. The fragments were fused to the minimal thymidine kinase promoter (minTK). ΔC/EBP1+2 refers to mutations of the two half sites that interact with C/EBP proteins and ΔGC-box to the mutation of the SP binding site as described preciously [17]. **G**. HeLa cells were co-transfected with the -184 to +248 reporter gene construct and C/EBPα and SMAD3 as indicated. For control the pGL2 reporter was measured. **H**. U937 cells were treated for the indicated times with TGFβ1. The cells were then fixed in formaldehyde, lysed and the DNA fragmented. The binding of SMAD3 to the *MAD1 *promoter was measured after specific immunoprecipiation and analysis of the promoter region using the primers given in figure 4B (P and P'). For control an irrelevant promoter, *MYOD1*, was used. The binding is given as % of input control.

Next we evaluated whether SMAD proteins are involved in activating the *MAD1 *promoter by using the -1282 to +248 *MAD1 *promoter reporter gene construct. This reporter was stimulated by a combination of SMAD2, 3, and 4 but the activity of these factors was not enhanced by coexpressing a constitutive active TGFβRI (TGFβRca) (Figure 6A). All these constructs however were active since a SMAD binding element reporter (SBE-luc) was strongly activated by SMADs and TGFβRca (Figure 6B and 6C). In the absence of exogenous SMAD proteins the TGFβRca was unable to significantly activate *MAD1 *promoter reporter constructs (Figure 6D). We further evaluated which SMAD protein(s) stimulated the *MAD1 *promoter reporter. We found by testing all combinations that only SMAD3 was stimulatory (data not shown). The SMAD3 responsive region was mapped to the promoter fragment that contains the two C/EBP half sites and one SP binding site, i.e. GC box1 (Figure 6E, for the positions of the binding sites see Figure 2A). These response elements appeared to be relevant because mutation of these sites in a reporter containing the -184 to -58 *MAD1 *promoter fragment upstream of the minimal thymidine kinase promoter (minTK) resulted in almost complete loss of SMAD3 responsiveness (Figure 6F). Consistent with this, C/EBPα and SMAD3 cooperated on the -184 *MAD1 *promoter reporter (Figure 6G). Finally we addressed whether SMAD3 interacted with the *MAD1 *promoter. Indeed we found that SMAD3 was bound to the *MAD1 *promoter but not to an irrelevant promoter (*MYOD1*) (Figure 6F). However stimulation of the U937 cells with TGFβ did not alter significantly the interaction of SMAD3 with the promoter. Together these findings demonstrate that SMAD3 functions as an activating transcription factor for the *MAD1 *promoter. The lack of regulation by coexpressing SMAD3 with TGFβRca as measured by reporter gene assays may be due to insufficient chromatin formation on the transfected DNA and/or additional important signaling compounds are missing.

### TGFβ1 stimulates Ser2 phosphorylation of Pol II

**Figure 7:**
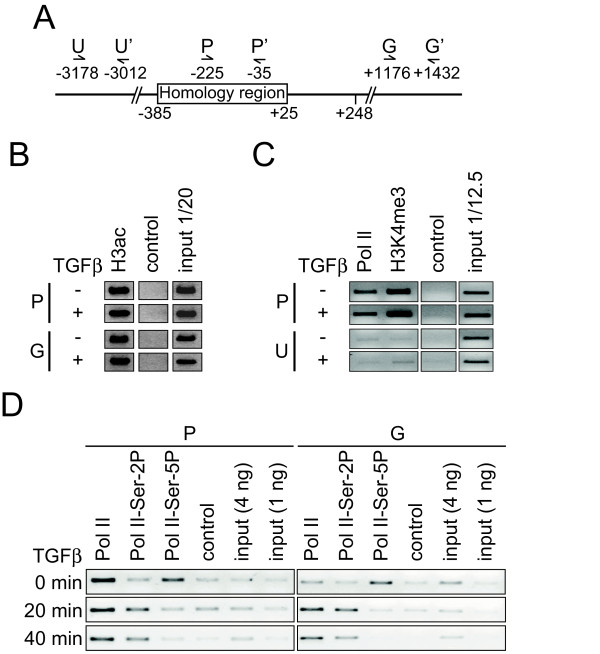
**Histone modifications and Pol II loading at the *MAD1 *promoter**. **A**. Summary of primer sets used for ChIP PCR relative to the major transcriptional start site within the *MAD1 *gene. **B**. and **C**. Proliferating U937 cells were treated with or without 2.5 ng/ml TGFβ1 for 1.5 h. ChIP experiments were performed with the indicated antibodies and primers. For control the immunoprecipitations were performed with Protein A Sepharose beads only. Signals obtained from input DNA, given as part of the immunoprecipitated material, are shown for comparison. **D**. U937 cells were treated with or without 2.5 ng/ml TGFβ1 for 20 and 40 min prior to cross-linking. For ChIP, total Pol II and Ser-2 and Ser-5 phosphorylated Pol II was immunoprecipitated. Loading and distribution of the different Pol II isoforms were examined on the *MAD1 *gene with the indicated primers.

To further evaluate how the *MAD1 *promoter is activated, we analyzed acetylation of histone H3 (H3ac) and trimethylation at Lys 4 of histone H3 (H3K4me3) before and after TGFβ1 stimulation. Both are marks for active promoters. We observed H3ac throughout the locus and H3K4me3 at the promoter, however, none of these marks was significantly modified by TGFβ1 stimulation (Figure 7A-C). These findings suggest that the *MAD1 *promoter is in an open configuration, similar to what has been observed recently for many promoters of regulated genes [29-31]. This is supported by our previous studies using nucleosomal mapping demonstrating open chromatin at the *MAD1 *proximal promoter [17]. Consistent with an open configuration is our observation that polymerase II (Pol II) occupied the *MAD1 *promoter constitutively (Figure 7D). Pol II was also detected in the gene body, where its binding increased in response to TGFβ1 treatment (Figure 7D). A key step in activating transcription is the differential phosphorylation of Pol II [32,33]. It is phosphorylated at Ser-5 of its C-terminal domain (CTD), a modification that defines a preactivation state. Upon stimulation, Pol II becomes phosphorylated at Ser-2 of the CTD, which coincides with elongating polymerase. Therefore we addressed whether phosphorylation at Ser-5 and Ser-2 was altered in response to TGFβ1. Indeed we observed an increase in Ser-2 phosphorylation upon TGFβ1 stimulation and a concomitant decrease of Ser-5 phosphorylation of Pol II both at the promoter and in the gene body (Figure 7D). Thus TGFβ1 regulates Pol II phosphorylation and activity.

## Conclusions

We observed that C/EBP and SP transcription factors bind constitutively to the proximal *MAD1 *promoter. In addition SMAD3, a factor typically activated by TGFβ signaling, also was found constitutively on the *MAD1 *promoter, despite the fact that no obvious binding sites for SMAD proteins are found. While the GC boxes are consensus binding sites for SP1, the proposed CCAAT boxes are deviating considerably from C/EBP consensus sequences. In fact, both elements that were identified functionally, represent only half sites [17]. Consistent with this interpretation, these DNA elements do not bind efficiently C/EBP homodimers in EMSA experiments in vitro. Surprisingly substantial binding was only measurable with C/EBPα/β heterodimers in these EMSA experiments. Nevertheless both factors were able to stimulate *MAD1 *promoter reporter genes. We did however not observe a strong synergistic activation by the two proteins, possibly due to abundant endogenous C/EBP factors (data not shown). We suggest that C/EBP and SP transcription factors form a platform for incoming signals as exemplified by G-CSF [17] and possibly TGFβ1. In the case of G-CSF, STAT3 is recruited by C/EBPs, requiring MAPK signaling. Our new findings suggest that TGFβ1 signaling activates SMAD proteins and stimulates MAPK signaling. The activation of MAPK might be a common pathway that controls at least in part *MAD1 *expression. Consistent with this interpretation, SMAD3 cooperated with C/EBP proteins to activate *MAD1 *promoter reporter genes. The finding that SMAD3 was bound to the *MAD1 *promoter suggests that SMAD3 is directly recruited to the *MAD1 *promoter by binding to C/EBPs or C/EBP associated factors. Because the GC box was also relevant, we propose that a large transcription factor/cofactor complex interacts with the identified promoter proximal region, including SMAD3. However, we point out that we cannot exclude direct binding of SMAD3 to the *MAD1 *promoter. Although no obvious binding sites could be detected, SMAD binding sites are rather short and leave the possibility open that SMAD3 forms a dimeric or multimeric complex with other factors, in which SMAD3 might bind directly to DNA.

The signals that are integrated at the proximal *MAD1 *promoter translate into the activation of Pol II as measured by its progression into the gene body and the concomitant change in the phosphorylation of the C-terminal domain of Pol II. This is consistent with recent observations on many genes, which have provided evidence that Pol II phosphorylated at Ser-5 is located at the promoter in a preactivated or paused mode. The switch to Ser-2 phosphorylation, possibly by the recruitment and activation of the P-TEFb kinase CDK9, results in the activation and promoter clearance of Pol II [34]. Thus this represents a situation as it is now becoming evident at many different promoters that are being studied in detail. It is worth noting that Pol II was found to be associated with the *MAD1 *promoter prior to stimulation with cytokines. Thus at least in U937 tumor cells, the *MAD1 *promoter is preoccupied by Pol II and thus allows for rapid activation by multiple signals. It will now be of interest to specifically dissect how different cytokines use the C/EBP-SP transcription factor platform to activate the paused Pol II.

## Methods

### Reporter gene construct and expression vectors

The cloning of *MAD1 *promoter reporter gene constructs has been reported previously [17]. Descriptions of pEQ176-ß-galactosidase, pCB6^+^-C/EBPα, and pCB6^+^-C/EBPβ are found in [28,35]; pCDNA3+-C/EBPε was obtained from A. Friedman [36]; pCL-neo-HA-SP1 and pCI-neo-HA-SP1-N (a dominant negative form of SP1) were provided by H. Rotheneder [37].

### Cell culture and treatment

HEK293 (ATCC CRL-1573) and HeLa (ATCC CCL-2) cells were cultured in DMEM (Gibco) with 10% fetal calf serum (Gibco) and penicillin/streptomycin (Seromed). U937 (ATCC CRL-1593.2) promyelocytes were grown in RPMI 1640 (Gibco) with 10% fetal calf serum and penicillin/streptomycin. All cells were cultured at 37°C and 5% CO_2_. U937 cells were treated with TGFβ1 (Peprotec) at a concentration of 2.5 ng/ml and with 5 μM SB505124 (Sigma-Aldrich) as indicated. Proliferation and viability of U937 cells were analyzed using Trypan Blue staining and the CASY cell counting system (Innovatis).

### Transient transfection and luciferase assay

Transient transfection of HEK293 and HeLa cells were performed using the calcium phosphate co-precipitation method as described previously [38]. HeLa cell co-transfected with pSuper-sh-C/EBPβ were harvested 72 hours post-transfection. For luciferase assays HeLa cells were co-transfected overnight with a total amount of 3-5 μg plasmid DNA and cultured for 48 hrs under normal growth conditions prior to harvesting. Luciferase activity was measured using a bioluminator (ELISA-Reader Victor2). The relative luciferase activity was normalized to the β-galactosidase activity. All experiments were performed in duplicates or triplicates with at least three independent replicates.

The online program siDirect (genomics.jp/sidirect) was used to design shRNA oligonucleotides targeting the *C/EBPβ *mRNA and the resulting sequences were analyzed via the BLAST algorithm. The hybridized oligonucleotides were cloned into the pSuper vector (obtained from R. Bernards) linearised with *Bgl*II and *Hin*dIII [39].

Sense: 5'GATCCCCAGCACAGCGACGAGTACAATCTTCAAGAGAGATTGTACTCGTCGCTGTGCTTTTTTGGAAA;

Antisense: 5'AGCTTTTCCAAAAAAGCACAGCGACGAGTACAATCTCTCTTGAAGATTGTACTCGTCGCTGTGCTGGG.

### RNA preparation and quantitative RT-PCR

The RNAeasy Mini Kit (Qiagen) was used for total RNA extraction, according to the manufacturer's instruction and residual genomic DNA was removed by DNase (Qiagen) digestion. 1 μg total RNA was reverse transcribed into cDNA using the Transcriptor First Strand cDNA Synthesis Kit (Roche) and analyzed by quantitative real time PCR using a LightCycler (Roche). The real time PCR reactions were performed with the SYBRgreen Ready Mix (Qiagen) and the following primer pairs: *MAD1 *QantiTect primer assay (Qiagen) and *β-GLUCURONIDASE*-f 5'-CTCATTTGGAATTTTGCCGATT-3', *β-GLUCURONIDASE*-r 5'-CCGAGTGAAGATCCCCTTTTTA-3'. The relative quantification of *MAD1 *mRNA was calculated by the comparative CT method and normalized to *β-GLUCURONIDASE *using the Software RelQuant.

### Chromatin immunoprecipitation (ChIP) assay and RE-ChIP assay

ChIP assays were performed as described previously [9]. U937 cells were grown in a spinner flask to a maximal density of 10^6 ^cells/ml. Following TGFβ1 treatment 5-2.5 × 10^7 ^cells/ml per IP were harvested. For immunoprecipitation 2 μg of the following antibodies were used: H3ac (06-599, Upstate); H3K4me3 (8580-50, Abcam); Pol II N20 (SC-899, Santa Cruz Biotechnology); Pol II CTD phosphoserine 2 H5 (MMS-129R, Covance); Pol II CTD phosphoserine 5 H14 (MMS-134R, Covance), C/EBPα 14AA (SC-61, Santa Cruz Biotechnology); C/EBPβ C19 (SC-150, Santa Cruz Biotechnology), SP1 PEP2 (SC-59, Santa Cruz Biotechnology), SP1 (07-124, Upstate), Cytochrome C (SC-7159, Santa Cruz Biotechnology), SMAD3 (ab28379 Abcam). In addition SP1-specific antibodies were obtained from G. Suske [40]. The following primer pairs were used for PCR analysis of the *MAD1 *gene:

U: 5'CCTCTTAATATACTGTCCTATGC-3';

U': 5'GTCACAGCTCTCCAGAAATAGAAG-3';

P: 5'AGTTGCGAATCCTGTCACCA-3';

P': 5'TTCTCTTGACAGGCCAGCTT-3';

G: 5'-ATATTGTAGGTGACACAAACTGC-3';

G': 5'-ATCTCACTTGAAGCTTCCACAG-3'

MYOD1: 5'-CCGCCGCTTTCCTTAACCACAAAT-3'

MYOD1': 5'-GTAGATAGCAAAGTGCTGGCAGTC-3'

For Re-ChIP assays the first immunoprecipitation was performed as above. Then the samples were washed once in ChIP RIPA buffer (150 mM NaCl, 10 mM Tris-HCl pH 7.5, 1 mM EDTA, 1% NP40, 0.1% DOC, 0.1% SDS, 0.5% aprotinin) and the protein-DNA complexes solubilized in release buffer (1% SDS, 10 mM DTT, TE-buffer pH 7.5). The beads were incubated at 37°C for 30 min. To the supernatant 4 volumes of RIPA-SDS (150 mM NaCl, 10 mM Tris pH 7.5, 1% NP40, 1% DOC, 0.5% aprotinin, 1 mM EDTA, 1 mM iodoacetamid) were added to perform the second immunoprecipitation.

### Electrophoretic mobility shift assay (EMSA)

The following oligonucleotides were γ^32^P-ATP radiolableled and used in EMSAs:

CCAAT-b1 f: 5'AGCCCTCTCCCAATCGCACAAG3';

CCAAT-b1 r: 5'CTTGTGCGATTGGGAGAGGGCT-3';

NE-CCAAT f: 5'-TCGAGATGGGGCAATAT-3';

NE-CCAAT r: 5'-ATATTGCCCCATCTCGA-3';

GC box1 f: 5'-AAGTGTAGGGGCGGGGCATTCT-3';

GC box1 r: 5'-AGAATGCCCCGCCCCTACACTT-3'.

HEK293 whole cell extracts were prepared on ice in Frackelton-lysis buffer (10 mM Tris-HCL pH 7.05, 50 mM NaCl, 30 mM Na4P2O7, 50 mM NaF, 5 μM ZnCl2, 1% (v/v) Triton X-100, 10% (v/v) glycerol, 100 μM Na3VO4, 150 μM benzamidin, 0.025 U/ml α-macroglobulin, 2.5 μg/ml leupeptin, 14 μg/ml aprotinin). Whole cell extracts were incubated with the radiolabeled oligonucleotides at 30°C for 30 min and then subjected to electrophoresis as described previously [41]. In brief, for supershift assays antibodies or equivalent amounts of control antibodies or BSA were added and incubated on ice for 10 min, prior to oligonucleotide addition. The protein-DNA complexes were separated on a 4.5% polyacrylamide gel containing 7.5% glycerol in 0.25-fold TBE (20 mM Tris base, 20 mM boric acid, 0.5 mM EDTA, pH 8) at 20 V/cm for 4 h. Gels were fixed in 10% methanol, 10% acetic acid, and 80% water for 1 h, dried, and autoradiographed. The following antibodies were used in EMSAs: C/EBPα 14AA (SC-61, Santa Cruz Biotechnology); C/EBPβ C19 (SC-150, Santa Cruz Biotechnology); SP1 PEP2 (SC-59, Santa Cruz Biotechnology), SP1 (07-124, Upstate), SP3 D-20 (SC-644, Santa Cruz Biotechnology), Cytochrom C (SC-7159, Santa Cruz Biotechnology).

### Western blotting

To generate highly concentrated U937 whole cell extracts (10^7 ^cells/preparation), U937 cells were lysed in 20 - 30 μl FT Lysis buffer (600 mM KCL, 20 mM Tris-Cl pH 7.8, 20% glycerol, 10 μg/ml leupeptin, 10 μg/ml pepstatin A, 14 μg/ml, aprotinin, 0.4 mg/ml, Pefabloc) by pipeting up and down as described previously [42]. The freeze-thaw cycles in liquid nitrogen were repeated five times. The thawed lysates were incubated with 250 U Benzonase (Merck) at RT for 10 min. Whole cell extracts were resolved by SDS-Page and transferred onto nitrocellulose membranes, probed with MAD1 C19 (SC-222, Santa Cruz Biotechnology), α-Tubulin (B-5-1-2, T-5168, Sigma), or C/EBPβ C19 (SC-150, Santa Cruz Biotechnology) antibodies followed by horseradish peroxidase (HRP)-labeled secondary antibody. Detection was performed with the either chemiluminescence ECL kit (Pierce) or SuperSignal West Femto Maximum Sensitivity Substrate (Pierce).

## List of abbreviations

C/EBP: CCAAT-enhancer-binding protein; ChIP: Chromatin immunoprecipitation; CTD: Carboxy-terminal domain; DMSO: Dimethyl sulfoxide; EMSA: Electrophoretic mobility shift assay; G-CSF: Granulocyte colony stimulationg factor; H3ac: acetylated histone 3; H3K4me3: trimethylated lysine 4 of histone 3; MAX: MYC-associated factor x; MYC: Myelocytomatosis viral oncogene; PTEN: Phosphatase and tensin homologue deleted on chromosome ten; Pol: Polymerase; Ser: Serine; SMAD3: Mothers against decapentaplegic homolog 3; SP: Specific protein; SPdn: Specific protein dominate negative; TGF: Transforming growth factor; TGFR: Transforming growth factor receptor; TPA:12-O-tetradecanoylphorbol-13-acetat.

## Competing interests

The authors declare that they have no competing interests.

## Authors' contributions

NH and BL designed the experiments. NH, KJ, and CC performed the experiments. NH, CC, and BL evaluated the data and wrote the manuscript. All authors read and approved the final manuscript.
